# *B. thetaiotaomicron*-derived acetic acid modulate immune microenvironment and tumor growth in hepatocellular carcinoma

**DOI:** 10.1080/19490976.2023.2297846

**Published:** 2024-01-25

**Authors:** Hongbin Ma, Liang Yang, Yingchao Liang, Fenghua Liu, Jinxiang Hu, Rui Zhang, Yong Li, Lei Yuan, Feiling Feng

**Affiliations:** a Department of Radiotherapy, Shanghai Eastern Hepatobiliary Surgery Hospital, Naval Medical University, Shanghai, People’s Republic of China; b Department of Radiation Center, Shanghai First Maternity and Infant Hospital, Tongji University School of Medicine, Shanghai, China; c Shanghai KR Pharmtech, Inc. Ltd, Shanghai, China; d Department of Hepatobiliary Surgery, Quzhou People’s Hospital, Quzhou, Zhejiang, China; e Department of Biliary Tract Surgery I, Shanghai Eastern Hepatobiliary Surgery Hospital, Naval Medical University, Shanghai, People’s Republic of China

**Keywords:** *B. thetaiotaomicron*, Hepatocellular carcinoma, epigenetic regulation, immune microenvironment modification

## Abstract

Hepatocellular carcinoma (HCC) is a leading cause of cancer-related deaths worldwide, and emerging evidence suggests that the gut microbiota may play a role in its development and progression. In this study, the association between *B. thetaiotaomicron*, a gut microbiota species, and HCC recurrence, as well as patient clinical outcomes, was investigated. It was observed that *B. thetaiotaomicron*-derived acetic acid has the potential to modulate the polarization of **pro-pro-inflammatory macrophagess**, which promotes the function of cytotoxic CD8+ T cells. The increased biosynthesis of fatty acids was implicated in the modulation of **pro-inflammatory macrophages** polarization by *B. thetaiotaomicron*-derived acetic acid. Furthermore, *B. thetaiotaomicron*-derived acetic acid was found to facilitate the transcription of ACC1, a key enzyme involved in fatty acid biosynthesis, through histone acetylation modification in the ACC1 promoter region. Curcumin, an acetylation modification inhibitor, significantly blocked the inhibitory effects of *B. thetaiotaomicron* and acetic acid on HCC tumor growth. These findings highlight the potential role of gut microbiota-derived acetic acid in HCC recurrence and patient clinical outcomes, and suggest a complex interplay between gut microbiota, immune modulation, fatty acid metabolism, and epigenetic regulation in the context of HCC development. Further research in this area may provide insights into novel strategies for HCC prevention and treatment by targeting the gut microbiota and its metabolites.

## 1. Introduction

Hepatocellular carcinoma (HCC), a primary liver cancer, remains a significant global health burden, accounting for a substantial number of cancer-related deaths. In addition, treatment options for advanced HCC are currently limited, posing significant challenges in managing this aggressive malignancy.^1^ HCC is the most common form of primary liver cancer, accounting for over 80% of all cases of liver malignancies.^2^ HCC arises from a complex interplay of various environmental factors, including viral infections such as hepatitis B virus (HBV) or hepatitis C virus (HCV), heavy alcohol consumption, and nonalcoholic fatty liver disease (NAFLD). These factors collectively contribute to chronic liver inflammation, which can ultimately culminate in the development of HCC. These investigations suggest that the development of chronic inflammation leading to HCC necessitates the presence of multiple predisposing factors, rather than a single factor acting in isolation.

A growing body of research has proposed a connection between the functions of gut epithelial barrier and gut microbiota with the development of hepatocellular carcinoma (HCC).^3,4^ In the intestinal tract, a solitary layer of epithelial cells acts as a protective barrier to prevent the passage of luminal contents, including gut microbes, into the body. Remarkably, individuals with chronic liver diseases, such as alcoholic hepatitis, cirrhosis, and hepatocellular carcinoma (HCC), display elevated levels of serum lipopolysaccharide (LPS) compared to healthy individuals, indicating enhanced permeability of the gut epithelial barrier.^5^ In an animal study, it was shown that inducing chemical disruption of the epithelial barrier in the liver promotes the development of tumors.^6^ Therefore, elevated gut permeability has been implicated in the process of tumorigenesis in individuals with chronic liver diseases. Additionally, dysfunction of the epithelial barrier can facilitate the translocation of metabolites from specific bacteria into the liver. These metabolites can then stimulate hepatic stellate cells (HSCs) to secrete inflammatory and tumor-promoting factors, thereby promoting the development of HCC in mice.^7^ Probiotics have the potential to modulate the gut microbiota and impact T-cell differentiation, which in turn can influence the microenvironment of HCC and the progression of tumors.^8^

Bacteroides thetaiotaomicron (*B. thetaiotaomicron*), belongings to the Bacteroidetes phylum, which is abundant in a healthy gastrointestinal (GI) tract, *B. thetaiotaomicron* holds significance as the first species to be sequenced.^9^ This prominent commensal species in the human gut plays a crucial role in various aspects of host well-being.Remarkably, *B. thetaiotaomicron* has been reported to contribute to the reduction of gut inflammation,^10^ reinforcement of innate immunity against pathogenic invasions,^11^ and processing of essential dietary nutrients.^12^ Additionally, it has exhibited excellent tolerance as a live biotherapeutic in patients with Crohn’s disease.^13^ The bacterium has emerged as a key player in comprehending and influencing the dynamics of the gut microbiota ecosystem.^14^As a dominant member among gut propionate producers, *B. thetaiotaomicron* represents a promising candidate for understanding and influencing the propionate pool in the host intestine. The propionate biosynthesis pathway has been identified in select human-associated commensal microbes and environmental prokaryotes. In recent years, an increasing number of functional and mechanistic studies on gut commensal bacteria have aided in identifying key metabolic pathways.^15^

In this study, we initially observed that Bacteroides is the genus with the most significant differences between the recurrent and non-recurrent groups in the fecal samples of hepatocellular carcinoma (HCC). Further investigation revealed that within Bacteroides, *B. thetaiotaomicron* is the species with the most significant differences. Therefore, we focused our study on *B. thetaiotaomicron*. We also found that two crucial enzymes responsible for acetic acid production in this bacterium show differences between the recurrent and non-recurrent groups. We conducted *in vitro* and *in vivo* experiments using mice to investigate the impact of *B. thetaiotaomicron* on hepatocellular tumor growth and explored potential correlations between tumor suppression, modulation of the tumor microenvironment, and alterations in the function of cytotoxic CD8+ T cells. Additionally, we conducted comprehensive molecular analyses to elucidate the underlying mechanisms through which *B. thetaiotaomicron* exerts its effects on HCC.

## 2. Results

### 2.1. B. thetaiotaomicron is associated with hepatocellular carcinoma cancer recurrence

**Figure 1.**
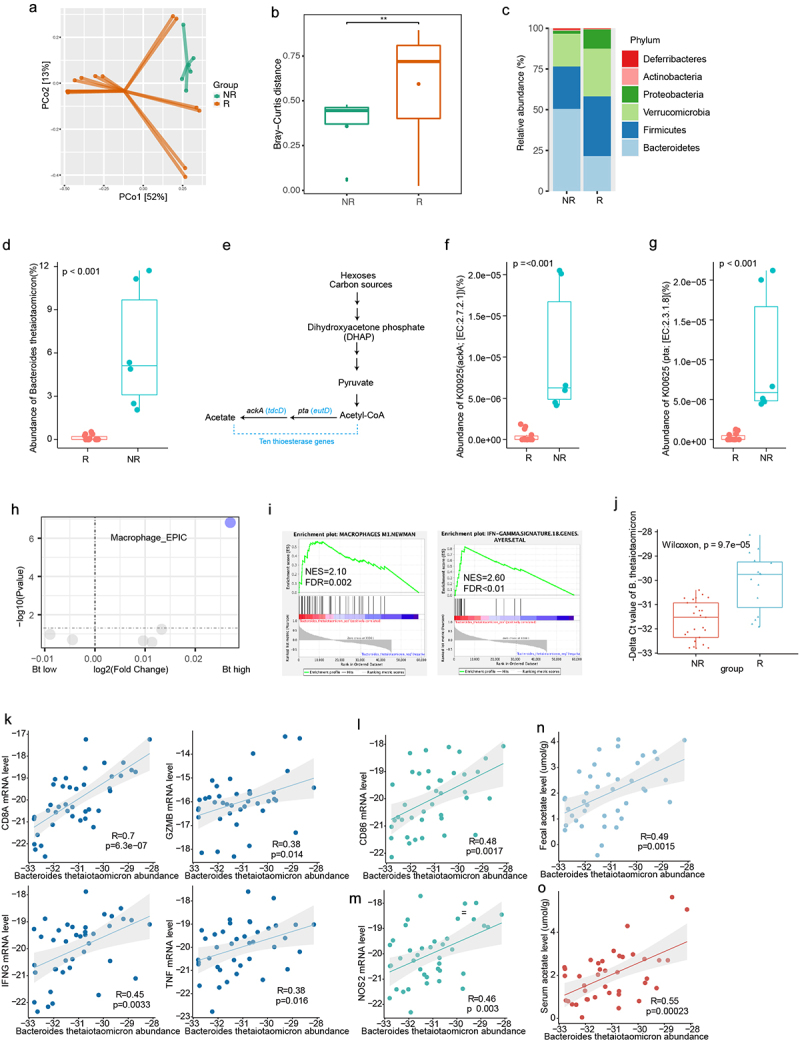
*B. thetaiotaomicron*-derived acetic acid correlated with recurrence in HCC. (a) Principal coordinate analysis (PCoA) plot of bacterial beta-diversity based on the Bray-Curtis dissimilarity according to the fecal metagenome sequencing data from HCC patients (recurrent group (*n* = 14) and non-recurrent group (*n* = 6). (b) Statistic result of Bray-Curtis distance according to the the fecal metagenome sequencing data of HCC patients (recurrent group (*n* = 14) and non-recurrent group (*n* = 6). (c) Histogram show the relative abundance between HCC patients (recurrent group (*n* = 14) and non-recurrent group (*n* = 6). (d) Box plot show the relative abundance of *B. thetaiotaomicron* between HCC patients (recurrent group (*n* = 14) and non-recurrent group (*n* = 6). (e) Schematic diagram depicting the enzyme in short chain fatty acid metabolism. (f) Box plot show the relative abundance of thioesterase gene ackA between HCC patients (recurrent group (*n* = 14) and non-recurrent group (*n* = 6). (g) Box plot show the relative abundance of thioesterase gene pta between HCC patients (recurrent group (*n* = 14) and non-recurrent group (*n* = 6). (h) Volcano plot revealed the differential abundant immune cell derived from EPIC. (i) Gene enrichment analysis base on the *B. thetaiotaomicron* abundance in HCC patients. (j) Real-time PCR detected the *B. thetaiotaomicron* abundance in HCC fecal samples. (k) Correlation between the expression of T cell effector marker and the relative abundance of *B. thetaiotaomicron*. (l) Correlation between the expression of macrophage marker and the relative abundance of *B. thetaiotaomicron*. (m) Correlation between the expression of macrophage marker and the relative abundance of *B. thetaiotaomicron*. (n) Correlation between the expression of the relative abundance of *B. thetaiotaomicron and* fecal acetate level. (o) Correlation between the expression of the relative abundance of *B. thetaiotaomicron and* serum acetate level.

The gut microbiota is involved in the occurrence and development of HCC. To investigate the recurrence of liver cancer after surgery, we analyzed the metagenomic data of 20 liver cancer patients(NR, non-recurrence *n* = 6; R, recurrence, *n* = 14). The results showed a significant difference in beta diversity between the recurrence and non-recurrence groups (Figure 1a,b). Further analysis at the phylum level revealed a significant decrease in the phylum Bacteroidetes in the recurrent group (Figure 1c, Figure S1a). At the species level, we found a significant enrichment of the species *Bacteroides thetaiotaomicron* (*B. thetaiotaomicron*) in the non-recurrent group (Figure 1 1d). Furthermore, we found that the use of the abundance of *B. thetaiotaomicron* yielded an area under the ROC curve of 85% The DCA plot indicated that using the abundance of Bacteroides thetaiotaomicron would provide a clinical net benefit (Figure S1b,c).

*B. thetaiotaomicron* contains thioesterases, which are closely related to the abundance of short-chain fatty acids (Figure 1e). We further analyzed the abundance of thioesterases between the two groups and found that the enzymes *ackA* and *pta*, which are associated with the production of acetic acid, were significantly enriched in the non-recurrence group (Figure 1f, g). We also examined the liver cancer tissues of these patients, performed RNA-seq, and used EPIC (Estimating the Proportions of Immune and Cancer cells) for tumor immune cell analysis. The results showed a significant enrichment of macrophages in the non-recurrence group (Figure 1h).

Further GSEA analysis showed that M1 macrophage and Macrophages M1_Newman pathway and activated IFN-gamma signature (Figure 1i) are more likely enriched in those HCC patients with high amount of *B. thetaiotaomicron* in their fecal sample. Further real time PCR data confirmed that the amount of *B. thetaiotaomicron* was indeed increased in non-recurrent HCC patients, compared with those recurrent patients (Figure 1j). Correlation analysis showed that *B. thetaiotaomicron* abundance positively correlated the RNA level of CD8A, GZMB, IFN-gamma and TNF, which represented the function of cytotoxic CD8+ T cells, which is essential immune cells for Immune surveillance of tumor cells (Figure 1k). We observed macrophage M1 polarization markers NOS2 was significantly correlated with *B. thetaiotaomicron* abundance (Figure 1l,m). It has been reported that *B. thetaiotaomicron* usually produces short chain fatty acid (SCFA).^16^ Next association analysis showed that the abundance of *B. thetaiotaomicron* positively correlated with different SCFAs, especially the level of acetate in the fecal samples (Figure 1n) and serum samples (Figure 1o). These correlation analysis indicated that *B. thetaiotaomicron* might secrete acetate, further promote M1 macrophage polarization and the function of cytotoxic CD8+ T cells, then finally inhibit tumor progression in HCC patients.

### 2.2. B. thetaiotaomicron-derived acetic acid participates in pro-inflammatory macrophages polarization and further promotes the function of cytotoxic CD8+ T cells

**Figure 2.**
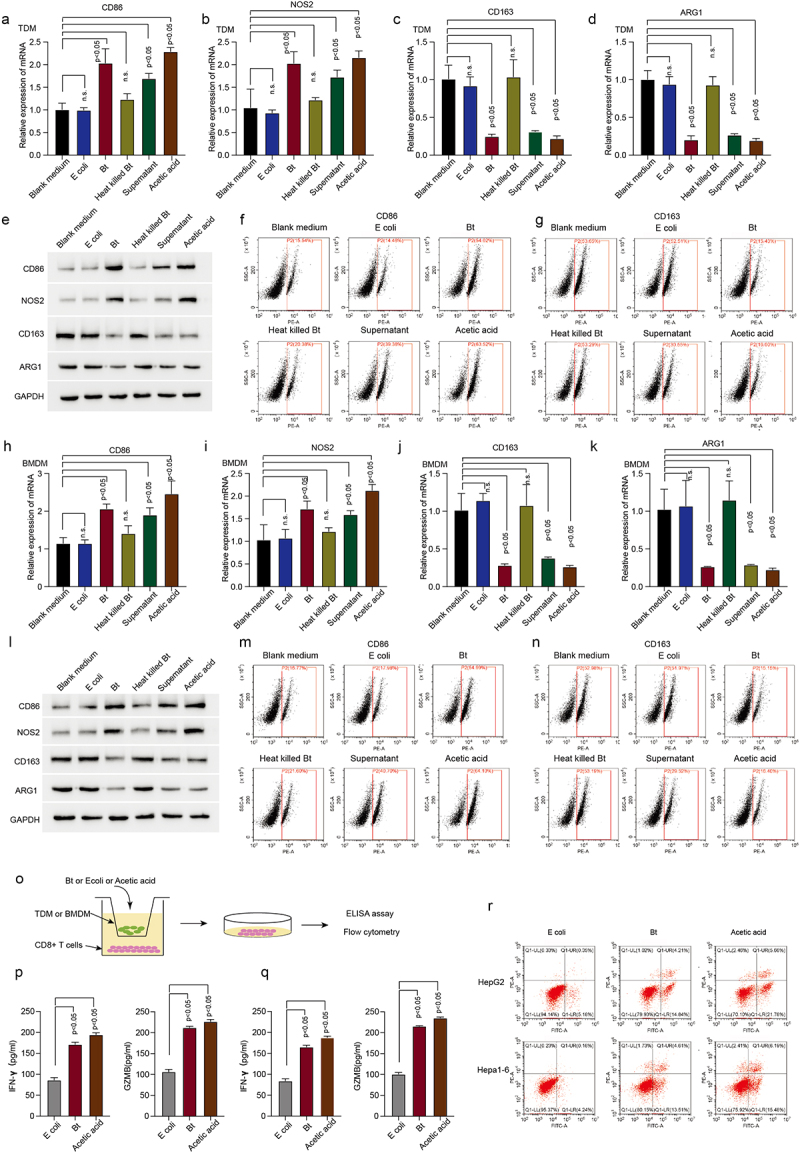
*B.thetaiotaomicron*-derived acetic acid participates in pro-inflammatory macrophages polarization and further promotes the function of cytotoxic CD8+ T cells. (a-d) real-time PCR was performed to measure the mRNA expression of CD86 (a) NOS2 (b) CD163 (c) and ARG1 (d) in TDM cells with indicated treatments. (*n* = 3). (e) Western-bolt was performed to measure the protein levels of CD86, NOS2, CD163 and ARG1 in TDM cells with indicated treatments. (*n* = 3). (f-g) flow cytometry was conducted to assess the levels of CD86 (f) and CD163 (g) in TDM cells with indicated treatments. (*n* = 3). (h-k) real-time PCR was performed to measure the mRNA expression of CD86 (h), NOS2 (i), CD163 (j) and ARG1 (k) in BMDM cells with indicated treatments. (*n* = 3). l. Western-bolt was performed to measure the protein levels of CD86, NOS2, CD163 and ARG1 in BMDM cells with indicated treatments. (*n* = 3). (m-n) flow cytometry was conducted to assess the levels of CD86 (m) and CD163 (n) in BMDM cells with indicated treatments. (*n* = 3). (o) The workflow of ELISA assay. (p) ELISA assay was conducted to measure IFN-gamma and GZMB levels in CD8+ T cells following co-culture with TDM cells that had been treated with *B. thetaiotaomicron* or acetic acid. (*n* = 3). (q) ELISA assay was conducted to measure IFN-gamma and GZMB levels in CD8+ T cells following co-culture with BMDM cells that had been treated with *B. thetaiotaomicron* or acetic acid. (*n* = 3). (r) Flow cytometry was performed to assess cytotoxicity of T cells against tumor cells after co-culture with macrophages treated with *B. thetaiotaomicron* and acetic acid. (*n* = 3).

To validate our high-throughput analysis and hypothesis, we used different fractions of *B. thetaiotaomicron* to treat human monocytes THP-1 derived macrophage (TDM). Real time PCR data showed that the mRNA level of CD86 (Figure 2a-2h) and NOS2 (Figure 2b-2i) (the markers of pro-inflammatory macrophages) was significantly increased in TDM and BMDM after *B. thetaiotaomicron* mixture, *B. thetaiotaomicron* culture medium, and acetic acid treatment, but not heat killed *B. thetaiotaomicron* or control bacteria *E.coli*. In addition, the mRNA level of CD163 (Figure 2c-2j) and ARG1 (Figure 2d-2k) (the markers of tumor-associated macrophage) was dramatically decreased in TDM and BMDM after *B. thetaiotaomicron* mixture, *B. thetaiotaomicron* culture medium, and acetic acid treatment, but not heat killed *B. thetaiotaomicron* or control bacteria *E.coli*. Western blot analysis data (Figure 2e-2l, Figure S2a, S2b) and Flow cytometry data (Figure 2f-2n), Figure S2c, S2d) further confirmed that the protein level of CD86 and NOS2 was significantly increased, while the protein level of CD163 and ARG1 was dramatically decreased in TDM and BMDM cells after *B. thetaiotaomicron* mixture, *B. thetaiotaomicron* culture medium, and acetic acid treatment. These data indicated that acetic acid, as the major component of *B. thetaiotaomicron* culture medium, may mediates the polarization of macrophages *in vitro*. To assess the potential impact of *B. thetaiotaomicron* on macrophage-mediated regulation of CD8+ T cell function, we co-cultured T cells with macrophages that had been treated with *B. thetaiotaomicron* or acetic acid (Figure 2o). The ELISA assay revealed a significant increase in IFN-gamma (Figure 2p) and GZMB (Figure 2q) levels in CD8+ T cells following co-culture with TDM and BMDM cells that had been treated with *B. thetaiotaomicron* or acetic acid. Furthermore, the co-culture of macrophages with *B. thetaiotaomicron* and acetic acid treatment resulted in a significant enhancement in the cytotoxicity of T cells against tumor cells (Figure 2r, Figure S2e).

**Figure 3.**
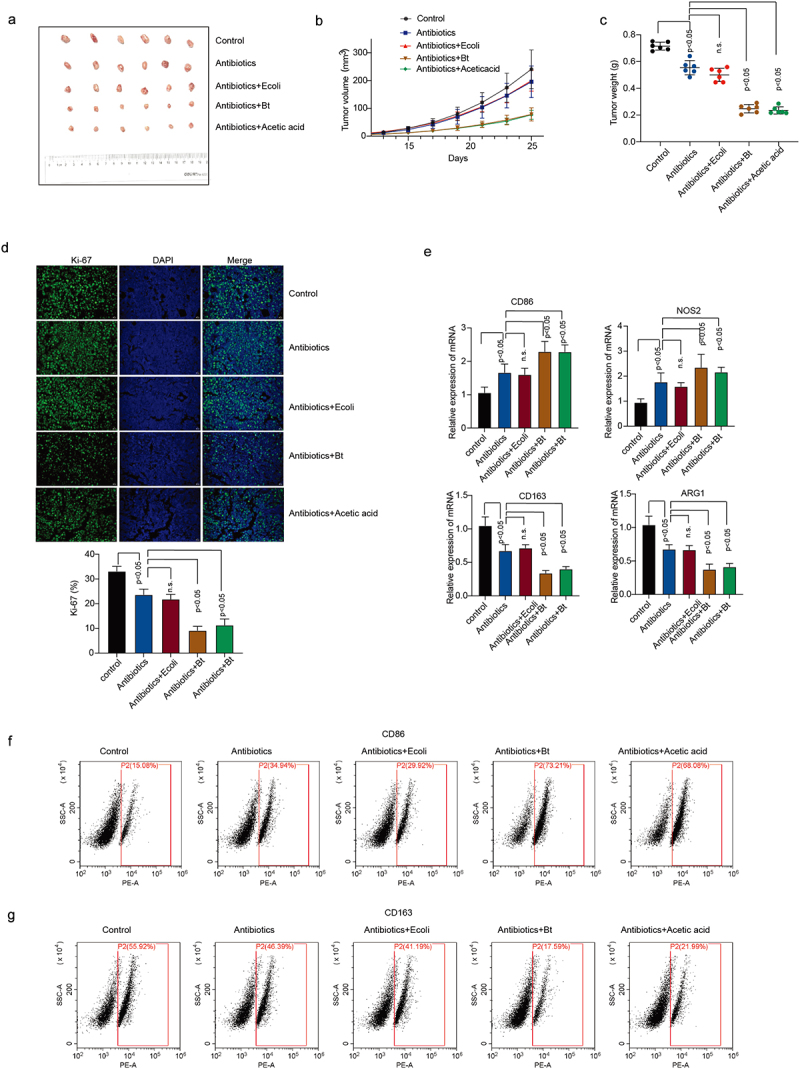
*B. thetaiotaomicron*-derived acetic acid participates in pro-inflammatory macrophages polarization in vivo. (a-c) tumor volumes (a-b) and tumor weights (c) were measured in mice bearing HCC cells treated with antibiotics, E coli, *B. thetaiotaomicron* or acetic acid. *n* = 6, nonparametric mann – Whitney test. (d) Immunofluorescence was performed to detect the expression of ki-67 in tumor models bearing HCC cells treated with antibiotics, E coli, *B. thetaiotaomicron* or acetic acid. (e) real-time PCR was performed to measure the mRNA expression of CD86, NOS2, CD163 and ARG1 in tumor models bearing HCC cells treated with antibiotics, ecoli, *B. thetaiotaomicron* or acetic acid. (*n* = 3). (f-g) flow cytometry was conducted to assess the levels of CD86 and CD163 in tumor models bearing HCC cells treated with antibiotics, E coli, *B. thetaiotaomicron* or acetic acid. (*n* = 3).

We also validated our findings *in vivo*. Administration of *B. thetaiotaomicron* and acetic acid significantly decreased the tumor growth (Figure 3a,b) and tumor weight (Figure 3c) in HCC mouse model. Ki67 Immunofluorescence data showed that *B. thetaiotaomicron* and acetic acid treatment significantly inhibited tumor cell proliferation in Hepa1–6 HCC mouse model (Figure 3d, Figure S3a). The treatment of HCC mouse tumor tissues with *B. thetaiotaomicron* and acetic acid resulted in upregulated expression of CD86 and NOS2, along with downregulated expression of CD163 and ARG1, as demonstrated by real-time PCR data (Figure 3e) and flow cytometry data (Figure 3f,g, Figure S3b). These findings suggest that *B. thetaiotaomicron* and acetic acid treatment may modulate the expression of immune-related genes, specifically CD86, NOS2, CD163, and ARG1, in HCC mouse tumor tissues, potentially influencing the immune microenvironment and tumor progression.

### 2.3. B. thetaiotaomicron-derived acetic acid might participate in M1 macrophage polarization via increasing the biosynthesis of fatty acid

**Figure 4.**
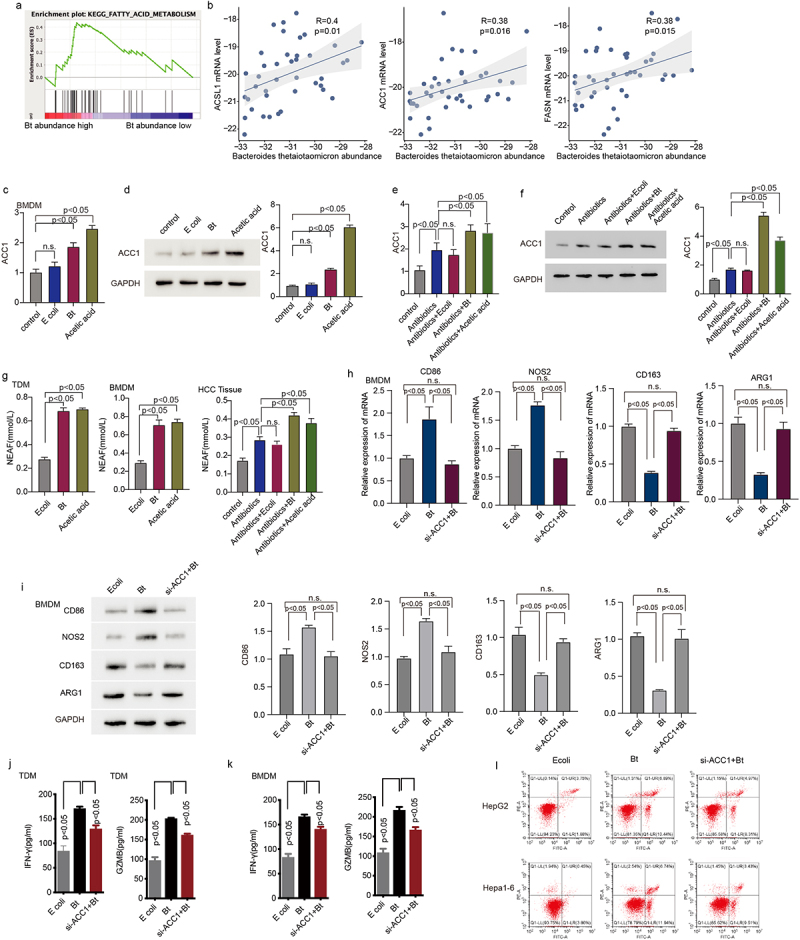
*B. thetaiotaomicron*-derived acetic acid might participate in M1 macrophage polarization via increasing the biosynthesis of fatty acid. (a) GSEA analysis was performed in HCC patients of cohort 1 with different level of *B. thetaiotaomicron*. (b) Correlation analysis was performed between ACSL1, ACC1 and FASN expression with *B. thetaiotaomicron* level in cohort 1. (c) The mRNA level level of ACSL1 was analyzed by real time PCR in BMDM after *B. thetaiotaomicron* and acetic acid treatment. (*n* = 3). (d) The protein level of ACSL1 was analyzed by Western blot in BMDM after *B. thetaiotaomicron* and acetic acid treatment. (*n* = 3). (e-f). The mRNA level (e) and protein level (f) of ACSL1, ACC1 and FASN was analyzed by real time PCR and Western blot in vivo after different treatment. (*n* = 3). (g) fatty acid production was evaluated in TDM-derived macrophage, BMDM and in HCC xenograft mouse model with different treatment. (*n* = 3). (h-i) the mRNA level (h) and protein level (i)of CD86, NOS2, CD163 and ARG1 was analyzed by real time PCR and Western blot in BMDM after different treatment. (j-k). The ELISA assay was performed to analyze the IFN-γ and GZMB level in CD8+ T cells after inoculated with TDM-derived macrophage (j) and BMDM (k) with different treatment. (*n* = 3). (l) Cytotoxic function of CD8+ T cells was evaluated in HCC cells after different treatment. (*n* = 3).

To dissect the mechanism of *B. thetaiotaomicron*-induced M1 macrophage, we performed GSEA (Gene Set Enrichment Analysis) analysis in our Cohort. GSEA analysis data showed that fatty acid metabolism is enriched in those HCC patients with high level of *B. thetaiotaomicron* (Figure 4a). Correlation analysis showed that the amount of *B. thetaiotaomicron* is positively correlated with the mRNA level of ACSL1, ACC1 and FASN (Figure 4b), which belong to major components of fatty acid synthesis pathway. Real time PCR and Western blot analysis data showed that the expression of ACC1, but not ACSL1 and FASN was significantly increased in TDM, BMDM and in HCC tumor tissue with *B. thetaiotaomicron* or acetic acid treatment (Figure S4a-d, Figure 4c-f). In addition, ELISA data showed that the production of fatty acid was significantly increased in TDM, BMDM and in HCC tumor tissue with *B. thetaiotaomicron* or acetic acid treatment (Figure 4g) as well. These data suggested that *B. thetaiotaomicron* and acetic acid-induced M1 macrophage polarization might depend on increase of fatty acid biosynthesis.

Additional real-time PCR and Western blot analysis revealed that specifically knocking down ACC1 significantly attenuated the *B. thetaiotaomicron*-induced upregulation of CD86 and NOS2, as well as the downregulation of CD163 and ARG1 in TDM (Figure S4e-f) and BMDM (Figure 4h,i) cells. We observed that ACC1 expression appears to be higher in HCC tissues compared to normal tissues, but this difference did not reach statistical significance (Figure S4g). Additionally, *B. thetaiotaomicron*-treated macrophages significantly enhanced the secretion of IFN-gamma and GZMB cytokines by CD8+ T cells (Figure 4j,k), as well as improved the T cell-mediated killing of tumor cells (Figure 4l). Notably, knockdown of ACC1 in THP-1-derived macrophages and BMDM cells dramatically blocked the observed increase in cytokine secretion and tumor cell killing of CD8+ T cells (Figure 4j-l). These data suggest that *B. thetaiotaomicron*-induced upregulation of ACC1 in macrophages may play a crucial role in enhancing T cell function, including cytokine secretion and tumor cell killing.

### 2.4. B. thetaiotaomicron-derived acetic acid facilitates the transcription of ACC1 by enhancing histone acetylation modification in the ACC1 promoter region

**Figure 5.**
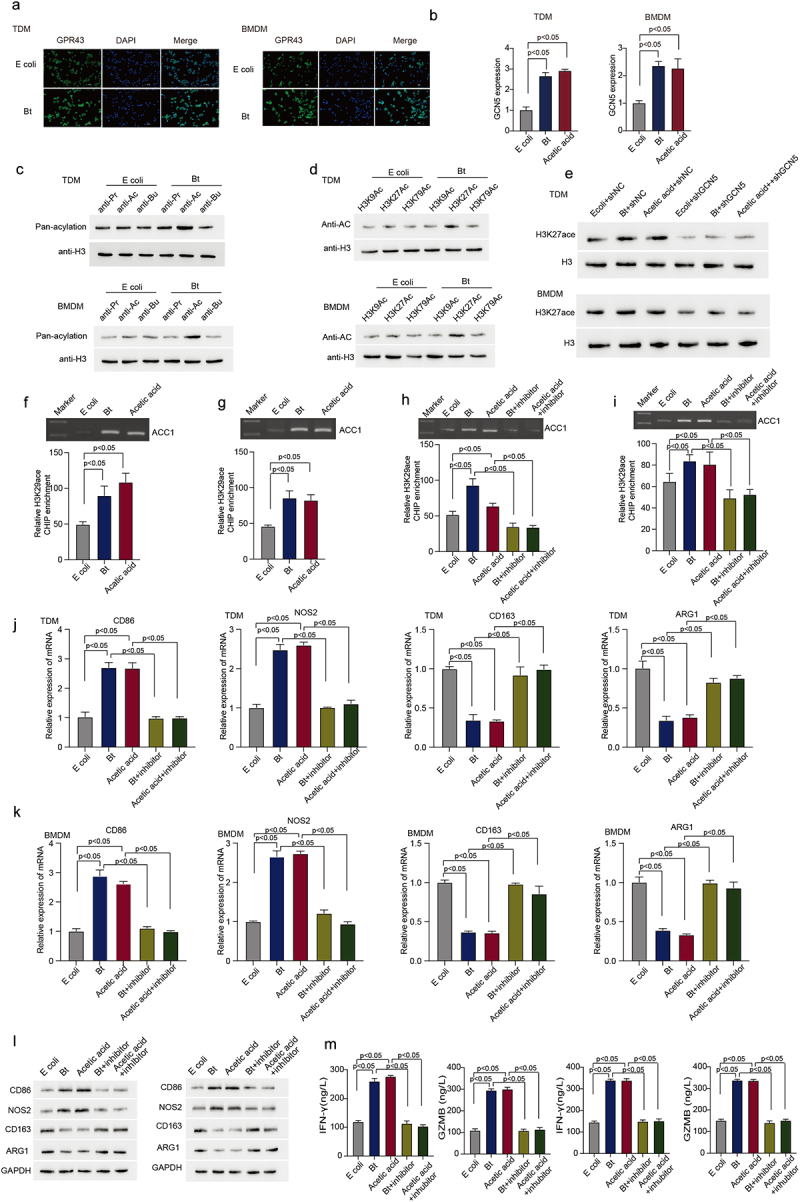
*B. thetaiotaomicron*-derived acetic acid facilitates the transcription of ACC1 by enhancing histone acetylation modification in the ACC1 promoter region. (a) Immunofluorescent assay was performed to analyzed the expression and location of GPR43 in TDM-derived macrophage and BMDM after E.Coli and *B. thetaiotaomicron* treatment. (b) The expression of GCN5 was analyzed in THP1-derived macrophage(TDM) and BMDM after *B. thetaiotaomicron* and acetic acid treatment. (*n* = 3).(c) Different modification of H3 histone was analyzed in TDM and BMDM after E.Coli and *B. thetaiotaomicron* treatment. (d) Acetylation level was analyzed at different site of H3 histone in TDM and BMDM after E.Coli and *B. thetaiotaomicron* treatment. (e) Western blot assay was performed to detect the level of H3K27ace in TDM and BMDM after different treatment. (f-g). The chip assay of H3K27ace at the promoter region of ACC1 was performed in TDM-derived macrophage (f) and BMDM (g)after *B. thetaiotaomicron* and acetic acid treatment. (*n* = 3). (h-i). The chip assay of H3K27ace at the promoter region of ACC1 was performed in TDM-derived macrophage (f) and BMDM (g) after different treatment. (*n* = 3). (j-k). The mRNA level of CD86, NOS2, CD163 and ARG1 was analyzed in TDM-derived macrophage (j) and BMDM (k) after different treatment. (*n* = 3). (l) The protein level of CD86, NOS2, CD163 and ARG1 was analyzed by Western blot in TDM-derived macrophage and BMDM after different treatment. (m) The ELISA assay was performed to analyze the IFN-γ and GZMB level in CD8+ T cells after inoculated with TDM-derived macrophage and BMDM with different treatment. (*n* = 3).

It has been reported that acetate supplement could induce a hyperacetylated state of histone H3 and affect the function of different cells.^17,18^ To detect whether *B. thetaiotaomicron*-derived acetic acid could affect the histone acetylation, we first detected the expression of G-protein coupled receptor 43 (GPR43), which could recognize short chain fatty acids, including acetic acid, and the expression of histone acetyltransferase (GCN5). Immunofluorescence data showed that *B. thetaiotaomicron* treatment significantly increased the expression of GPR43 (Figure 5a) and GCN5 (Figure 5b) in TDM and BMDM cells. Further Western blot data showed that only the acetylation level of H3 histone, but not other modification level was significantly increased in TDM (Figure 5c, Figure S5a) and BMDM (Figure 5c, Figure S5a) cells after *B. thetaiotaomicron* treatment. Western blot analyses of histone acetylation levels at various sites revealed a significant increase specifically in H3K27 acetylation, but not in other sites, in TDM (Figure 5d, Figure S5b) and BMDM (Figure 5d, Figure S5b) cells following treatment with *B. thetaiotaomicron*. In addition, knockdown of GCN5 effectively attenuated *B. thetaiotaomicron* and acetic acid-induced upregulation of H3K27 acetylation in TDM (Figure 5e, Figure S5c) and BMDM (Figure 5e, Figure S5c). Additionally, ChIP assay data demonstrated that treatment with *B. thetaiotaomicron* and acetic acid significantly increased the acetylation level of H3K27 at the promoter region of ACC1 (Figure 5f,g). Furthermore, treatment with curcumin, an acetylation modification inhibitor, significantly inhibited the enrichment of H3K27 acetylation at the ACC1 promoter region induced by *B. thetaiotaomicron* and acetic acid (Figure 5h,i). Additionally, the upregulation of CD86 and Nos2, as well as the downregulation of CD163 and ARG1, induced by *B. thetaiotaomicron* and acetic acid were markedly blocked after curcumin treatment in TDM and BMDM cells (Figure 5j-l, Figure S5d-e). Furthermore, the enhanced expression of GZMB and IFN-gamma cytokines in CD8+ T cells, induced by macrophages after *B. thetaiotaomicron* and acetic acid treatment, was significantly suppressed upon curcumin treatment (Figure 5m). These findings suggest that *B. thetaiotaomicron* and acetic acid-induced pro-inflammatory macrophages polarization and improved CD8+ T cell function may be mediated by increased transcription of ACC1, potentially through enhanced H3K27 acetylation at the ACC1 promoter region.

### 2.5. Acetylation modification inhibitors Attenuates Inhibitory Effect of B.thetaiotaomicron and Acetic Acid on HCC Tumor Growth

**Figure 6.**
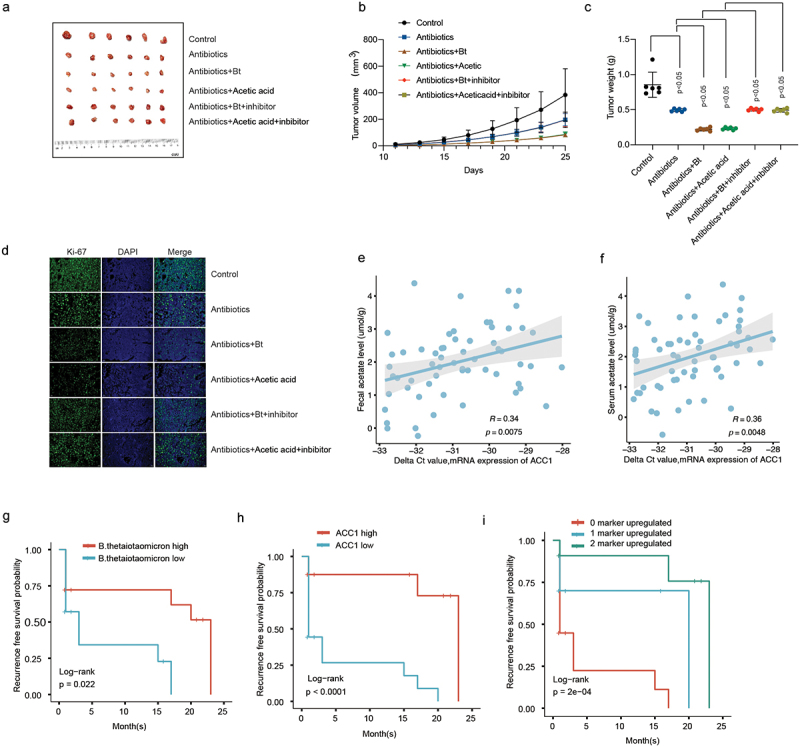
The inhibitory effect of *B. thetaiotaomicron* and acetic acid on the growth of HCC tumors and the prognostic significance of *B. thetaiotaomicron* abundance. (a) Representative image illustrating the inhibitory effect of *B. thetaiotaomicron* and acetic acid on HCC tumor growth in mice. (b) The inhibitory impact of *B. thetaiotaomicron* and acetic acid on HCC tumor volume. (c) The inhibitory effect of *B. thetaiotaomicron* and acetic acid on HCC tumor weight. (d) Representative image depicting the inhibitory effect of *B. thetaiotaomicron* and acetic acid on HCC through immunofluorescence in mice. (e) Correlation between the relative expression of ACC1 and the fecal acetate level(*n* = 60). (f) Correlation between the relative expression of ACC1 and the serum acetate level(*n* = 60). (g) Kaplan-meier curves showing the recurrence-free survival of patients with different abundance of *B. thetaiotaomicron*. (h) Kaplan-meier curves showing the recurrence-free survival of patients with different levels of ACC1. (i) kaplan-meier analysis of recurrence-free survival for HCC patients based on the number of upregulated molecular markers (*B. thetaiotaomicron* and ACC1) in cohort 2.

In vivo validation assay of curcumin and C646, two acetylation modification inhibitors, treatment demonstrated that they significantly attenuated the *B. thetaiotaomicron* and acetic acid-induced decrease in HCC tumor growth (Figure 6a,b, Figure S6a-b), tumor weight (Figure 6c), and tumor cell proliferation (Figure 6d) in HCC xenograft mouse model. We further examined the correlation between ACC1 and fecal acetate levels as well as serum acetate levels in HCC cohort 2(*N* = 60). We found a significant positive correlation between ACC1 and both fecal acetate levels (Figure 6e) and serum acetate levels (Figure 6f). Survival analysis revealed that *B. thetaiotaomicron* abundance, ACC1, and the combination of these two biomarkers could predict the prognosis of HCC (Figure g-i). These data highlighted the potential of curcumin and the association between ACC1 and *B. thetaiotaomicron* levels as promising targets for therapeutic interventions and prognostic evaluation in HCC.

## 3. Discussion

The association between *B. thetaiotaomicron* and hepatocellular carcinoma (HCC) recurrence, as well as patient clinical outcome, has been a subject of investigation in this study. It was observed that *B. thetaiotaomicron*-derived acetic acid has the potential to modulate the polarization of M1 macrophages, which in turn promotes the function of cytotoxic CD8+ T cells. Further analysis revealed that *B. thetaiotaomicron*-derived acetic acid may contribute to M1 macrophage polarization by increasing the biosynthesis of fatty acids. Moreover, it was observed that *B. thetaiotaomicron*-derived acetic acid facilitates the transcription of ACC1 by enhancing histone acetylation modification in the ACC1 promoter region. Notably, treatment with curcumin, an acetylation modification inhibitor, significantly blocked the inhibitory effects of *B. thetaiotaomicron* and acetic acid on HCC tumor growth.

The findings of this study shed light on the potential role of *B. thetaiotaomicron* and its derived acetic acid in HCC recurrence and patient clinical outcomes. The modulation of M1 macrophage polarization by *B. thetaiotaomicron*-derived acetic acid suggests that the gut microbiota may have an impact on the immune microenvironment of HCC. The promotion of cytotoxic CD8+ T cell function by M1 macrophages may indicate a potential mechanism by which *B. thetaiotaomicron*-derived acetic acid exerts its anti-tumor effects in HCC. This finding is in line with previous studies demonstrating that probiotic administration promotes a favorable shift of the gut microbial community toward beneficial bacteria, including Prevotella and Oscillibacter, which are known to reduce Th17 polarization and promote the differentiation of anti-inflammatory Treg/Tr1 cells in the gut, and further contribute to the suppression of hepatocellular carcinoma growth in mice.^8^

Another interesting observation from this study is the involvement of fatty acid biosynthesis in the modulation of M1 macrophage polarization by *B. thetaiotaomicron*-derived acetic acid. Fatty acids are important components of cell membranes and play crucial roles in cellular processes such as energy metabolism, inflammation, and immune response.^19–22^ The increased biosynthesis of fatty acids may contribute to the pro-inflammatory phenotype of M1 macrophages, which are known to exert anti-tumor activities. This suggests a potential link between gut microbiota-derived acetic acid, fatty acid metabolism, and immune modulation in the context of HCC.

Furthermore, the study provides insights into the epigenetic regulation of ACC1, a key enzyme involved in fatty acid biosynthesis, by *B. thetaiotaomicron*-derived acetic acid. The enhancement of histone acetylation modification in the ACC1 promoter region by acetic acid suggests that epigenetic mechanisms may be involved in the regulation of ACC1 expression. Epigenetic modifications, such as histone acetylation, can alter gene expression without changing the DNA sequence and have been implicated in various diseases, including cancer.^23–25^ The findings suggest a potential epigenetic mechanism by which *B. thetaiotaomicron*-derived acetic acid may modulate ACC1 expression and, consequently, fatty acid metabolism in the context of HCC.

The results of this study also highlight the inhibitory effects of curcumin and C646, two acetylation modification inhibitors,^26,27^ on the anti-tumor effects of *B. thetaiotaomicron* and acetic acid in HCC. Acetylation modification inhibitors has been widely studied for anti-cancer properties^28,29^ and their ability to block the effects of *B. thetaiotaomicron* and acetic acid suggests that acetylation modification may play a crucial role in the observed effects of *B. thetaiotaomicron*-derived acetic acid in HCC. This underscores the importance of epigenetic regulation in the context of gut microbiota-host interactions and HCC development.

This study has a limitation in that while the *in vitro* and preclinical findings offer valuable insights, their direct translation to human clinical settings may face challenges. Human responses to microbial metabolites can be influenced by various factors such as genetic diversity, diet, and lifestyle, which were not comprehensively considered in this research. Further investigations are required to substantiate the potential therapeutic use of *B. thetaiotaomicron* or acetic acid derived from *B. thetaiotaomicron* in HCC patients. Conducting clinical trials is imperative to evaluate the safety and effectiveness of interventions aimed at modulating the gut microbiota in individuals with HCC.

In conclusion, the findings of this study provide valuable insights into the potential role of *B. thetaiotaomicron* and its derived acetic acid in HCC recurrence and patient clinical outcomes. The modulation of M1 macrophage polarization, involvement of fatty acid biosynthesis, epigenetic regulation of ACC1, and the inhibitory effects of curcumin highlight the complexity of gut microbiota.

## 4. Methods

### 4.1. Patient specimens

HCC tissues were obtained from patients underwent surgery at Shanghai Eastern Hepatobiliary Surgery Hospital. The study protocol was approved by the ethics committee of Shanghai Eastern Hepatobiliary Surgery Hospital. Written informed consent was obtained from all participants in this study. All the researches were carried out in accordance with the provisions of the Declaration of Helsinki of 1975.

### 4.2. Cell culture and treatment

The human and mice HCC cell lines were purchased from American Type Culture Collection (ATCC). All the cell lines were cultured in the recommended growth medium supplemented with 10% fetal bovine serum in an atmosphere of 5% CO2 at 37°C.

### 4.3. Growth conditions of bacteria

The *B. thetaiotaomicron* was purchased from MINGZHOUBIO company with catalog number B80048. *B. thetaiotaomicron* was grown in BHIS broth supplemented with erythromycin (15 μg/mL), tetracycline (2.5 μg/mL), gentamycin (200 μg/mL), 5’-fluoro-2’-deoxyruidin (200 μg/mL), anhydrotetracycline (0.1 μg/mL), D-glucose, D-mannose, D-rhamnose, D-cellobiose, D-maltose (0.5% wt/vol), hemin from bovine (25 mg/L and 50 mg/L), porcine mucin extract (0.1% and 0. 5% wt/vol), bile extract from bovine and ovine at 0.5% unless indicated otherwise, DNase I (98 U/mL), or RNase1 (0.06 U/mL) when required. Cultures were incubated at 37°C in anaerobic-microaerophilic station.

### 4.4. Differentiation of THP-1 cells

To generate THP-1 derived macrophages (TDM), THP-1 cells (in 0.4 μm transwell insert for 6-well plate) were treated with 10 ng/ml phorbol 12-myristate 13-acetate (PMA) for 24 hours. Then, the cells were cultured with 25 ng/ml IL-4 or LPS for another 48 hours to generate THP-1-derived macrophages. Meanwhile, THP-1 cells were treated with 10 ng/ml phorbol 12-myristate 13-acetate (PMA) for 24 hours before further treatment (*B. thetaiotaomicron*, heat killed *B. thetaiotaomicron*, *B. thetaiotaomicron* culture medium or acetic acid).

### 4.5. Bone marrow-derived macrophage isolation and culture

Mouse bone marrow-derived macrophages (BMDMs) were isolated from male wild-type C57BL/6 mice, and cultured in Dulbecco’s Modified Eagle Medium (DMEM) supplemented with 10% FBS, 1% penicillin/streptomycin and 20 ng/mL macrophage colony-stimulating factor (M-CSF) for stimulation. Half volume of fresh grow medium was added after 3 days of culture. BMDMs were harvested for further treatment (*B. thetaiotaomicron*, heat killed *B. thetaiotaomicron*, *B. thetaiotaomicron* culture medium, acetic acid) and testing after 7 days of culture.

### 4.6. Cell apoptosis analysis with flow cytometry

Cells were collected and washed twice with PBS. After centrifuge, they were re-suspended (2×E6 in 400 ul of PBS). Annexin V (1 mg/ml) and propidium iodide (1 mg/ml) were used for the cell staining and herein flow cytometry analysis. Cells that were PI negative and Annexin V negative are considered healthy, PI negative and Annexin V positive are apoptotic, and both PI and Annexin V positive are considered necrotic.

### 4.7. Adenovirus construction

The shNC adenovirus, sh-ACC1 adenovirus were constructed by Obio Technology Company (Shanghai, China).

### 4.8. Total RNA extraction and real-time PCR

Total RNA was extracted from HCC tissues and cell lines using trizol reagent (Takara, Japan). Total RNA was reverse transcribed by PrimeScript RT Reagent Kit (Takara, Japan). Quantitative real-time PCR was performed using ABI reagent (Thermo Fisher Scientific, USA) by StepOnePlus real-time PCR system (Applied Biosystems, Foster City, CA). 2^−ΔΔCt^ method was used to quantify the relative expression levels. GAPDH was used as an internal control.

### 4.9. Detection of B. thetaiotaomicron

The primer sequence of *B. thetaiotaomicron* and the method for *B. thetaiotaomicron* detection were described previously.^30^ gDNA from each specimen was subjected to qPCR to determine the amounts of *B. thetaiotaomicron* by detecting the 16S genes. Each reaction contained 40 ng of gDNA and was assayed in triplicate in 10 μL reactions containing 1 × Power SYBR Green PCR Master Mix (Thermo Fisher Scientific, West Palm Beach, FL),0.4 μM each primer and was placed in a 96-well optical PCR plate. Primer sequences for *B. thetaiotaomicron* were 5’-GCAAACTGGAGATGGCGA-3’ and 5’-AAGGTTTGGTGAGCCGTTA-3’ (Tm 62.5°C). DNA extractions from pure cultures of *B. thetaiotaomicron* were used to generate standard curves using 7 serial dilutions ranging from 442 ng/ml to 4.42✖10^−4^ ng/ml and 235 mg/ml to 2.35✖10^−4^ ng/ml, respectively. Amplification and detection of DNA was performed with the ABI StepOne Plus Real-Time PCR System (Applied Biosystems, Foster City, CA) under the following reaction conditions: denaturation step (95°C for 30 sec), amplification step (35 cycles of 95°C for 10 s, 62.5°C for 15 s and 60°C for 30 s), and melting cycle (65–95°C 0.5°C increment 2–5 s/step).

### 4.10. Western blot

Total protein was extracted by RIPA lysis buffer (Beyotime, China) containing a protease inhibitor mixture (protease inhibitors; phosphatase inhibitors; PMSF; KangChen, Shanghai, China). The concentration of protein was quantified using BCA Protein Assay Kit (Thermo Fisher Scientific, West Palm Beach, FL). Briefly, extracted protein was separated by 10% SDS-polyacrylamide gels and then transferred to PVDF membranes (Biorad, Hercules, CA). After blocked with 5% BSA at room temperature for 2 hours, the membranes were incubated with primary antibody 4°C overnight. Next day the membranes were washed with TBST for five times and incubated with species-specific secondary antibodies for one hour at room temperature. Secondary antibodies were labeled with HRP. The ECL detection system was used for visualization. Antibodies against GAPDH acted as an internal control.

### 4.11. Immunofluorescence

Cells were rinsed twice with PBS and fixed with 4% PFA for 10 min, at room temperature. Blocking and permeabilization were performed with 2% BSA and 0.1% triton X-100 after washing three times with PBS to remove the fixative. The primary anti-KI67 and anti-GRP43 antibody were diluted and incubated with samples overnight at 4°C. After rinsing with PBS, the secondary antibody were added and incubated with the cells for 1 h at room temperature. DAPI were used for nuclear staining and samples were mounted in fluorescence antifade mounting media.

### 4.12. Fatty acid measurement

The FFA detection were followed the kit protocol. Breifly, 100 ul of standards or 100 ul of samples were added into appropriate wells, and PBS only for the blank control. Mix the well after adding 50 ul of the conjugate. Cover the plate and incubate for 1 h at 37. The concentration of fatty acids is directly proportional to the optical density at 570 nm.

### 4.13. Measurement of GZMB and IFN-γ concentration

Cells were seed into six-well plate and incubated with Ecoli or Bt. The culture medium was collected after incubation for 48 hours. The concentration of GZMB and IFN-γ in the supernatant of CD8+ cells were measured using Human Granzyme B ELISA Kit and Human IFN-γ ELISA Kit (Abcam, Cambridgeshire, UK) according to the manufacturer’s protocols. The absorbance was measured at 450 nm in a microplate reader. All the experiments were performed in triplicate.

### 4.14. Chromatin immunoprecipitation

Chromatin immunoprecipitation (ChIP) assays were conducted using the ChIP Assay Kit (Millipore, New Bedford, MA) according to the manufacturer’s protocols. Briefly, cells were cross-linked with formaldehyde and collected using SDS lysis buffer. The chromatin was sonicated to lengths between 200 and 800 bp. The DNA-protein complexes were pre-cleared with Protein A Agarose/Salmon DNA and then immunoprecipitated with antibodies. The co-precipitated DNAs were extracted using phenol/chloroform and subjected to real-time PCR analysis.

### 4.15. In vivo *xenograft model*

4-week-old male BALB/c nude mice were purchased from Experimental Animal Center of SIBS. HCC cells were injected into the right flank of mice subcutaneously to establish the HCC xenograft model. Two weeks after inoculation, the mice were injected with Bt or Ecoli by multipoint intratumoral injection every two days. Tumor volume (mm^3^ was assessed by the formula: Tumor volume (mm^3^ = longer diameter x shorter diameter^2^/2. All mice were maintained under specific pathogen-free conditions and used in accordance with the animal experimental guidelines set by the Institute of Animal Care and Use Committee. This study has been approved by the Institutional Animal Care and Use Committee of Shanghai Eastern Hepatobiliary Surgery Hospital.

### 4.16. GSEA analysis

Gene set enrichment analysis (GSEA) was performed to gain further insight into the biological pathways involved in HCC through Bt. FDR 0.25 is a well-established cutoff for the identification of biologically relevant genes. The gene sets showing FDR, 0.25, were considered enriched between the classes under comparison. Enrichment analysis applied the gene sets collection (c2.all.v5.0.sym- bols.gmt) from the Molecular Signatures Database – MsigDB.

### 4.17. Metagenomic analysis and of EPIC (estimating the Proportions of Immune and cancer cells) analysis

In this analysis, raw FASTQ files underwent a quality filtering step using the default parameters of the fast tool. For the taxonomic classification of bacteria, the remaining reads were processed using the mOTUs2 software, and the results were combined at various taxonomic levels using the R package phyloseq. Metagenomic sequencing data were subjected to quality control using Trimmomatic. Human host sequences were removed by aligning the reads to the human reference genome GRC37/hg19 with Bowtie2, and the filtered reads were further processed using Samtools. Functional profiling of the metagenomic data was performed using HUMAnN2 v2.8.1, and gene families were annotated using UniRef90 identifiers. The relative abundance of the gene families was normalized and regrouped using the “humann2_regroup_table” command to obtain KEGG Orthology (KO) terms.Additionally, for the analysis of tissue RNA sequencing (RNAseq) data, raw counts data were transformed into transcripts per million (TPM). Furthermore, the abundance of various immune cells within the tissue was calculated using the EPIC package, which can be found at this GitHub repository: https://github.com/GfellerLab/EPIC.

### 4.18. Statistical analysis

Statistical analyses were carried out using the program R (www.r-project.org). Data from at least 3 independent experiments conducted in triplicates were presented as the mean±SEM. Measurement data between two groups were performed using nonparametric Mann-Whitney test. Differences were considered to be significant with a value of *p* < 0.05.

## Supplementary Material

Supplemental Material

Figure S1.docx

Figure S3.docx

Figure S6.docx

Figure S4.docx

Supplementary Table 1.docx

Figure S5.docx
