# Supplementary figures and images for: B. thetaiotaomicron-derived acetic acid modulate immune microenvironment and tumor growth in hepatocellular carcinoma
Source: Gut Microbes. 2024 Jan 25;16(1):2297846. doi: 10.1080/19490976.2023.2297846 (PMC10813637; doi:10.1080/19490976.2023.2297846)

Figure S2

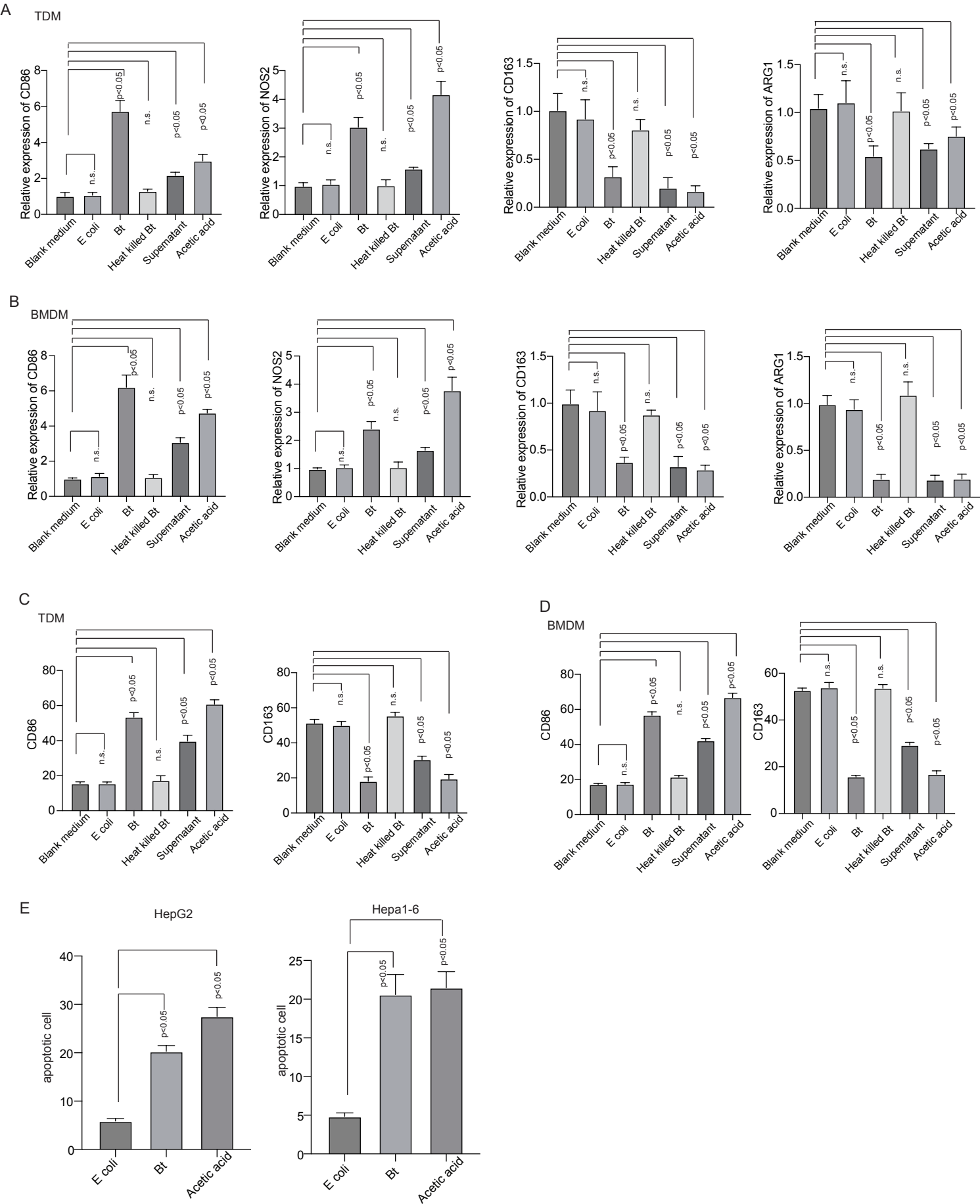

Supplement: Supplemental Material [file KGMI_A_2297846_SM9862.pdf]
