# Supplementary material for: B. thetaiotaomicron-derived acetic acid modulate immune microenvironment and tumor growth in hepatocellular carcinoma
Source: Gut Microbes. 2024 Jan 25;16(1):2297846. doi: 10.1080/19490976.2023.2297846 (PMC10813637; doi:10.1080/19490976.2023.2297846)
Supplement: Figure S1.docx [file KGMI_A_2297846_SM9034.docx]

Figure S1

A

Metagenomics data

Quantify the bacterial abundance by mOTUs

Differential test in Phylum level

Phylum of Bacteroidetes

Most enriched species in non-recurrence group

Bacteroides thetaiotaomicron

B C

**Confidence intervals**

B. thetaiotaomicron abundance All

None

80

100

0.6

0.0 0.1 0.2 0.3 0.4

AUC: 85.3% (72.3%-98.3%)

Sensitivity (%)

20

40

60

Net Benefit

0.0

0.2

0.4

Threshold probability

0

100 80 60 40 20 0

Specificity (%)
