# Supplementary material for: B. thetaiotaomicron-derived acetic acid modulate immune microenvironment and tumor growth in hepatocellular carcinoma
Source: Gut Microbes. 2024 Jan 25;16(1):2297846. doi: 10.1080/19490976.2023.2297846 (PMC10813637; doi:10.1080/19490976.2023.2297846)
Supplement: Figure S3.docx [file KGMI_A_2297846_SM9033.docx]

# Figure S3

A

CD86

p<0.05

p<0.05

# B

80


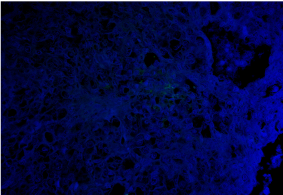

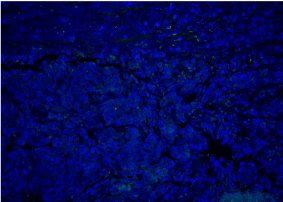

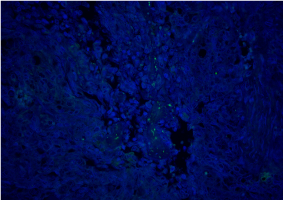

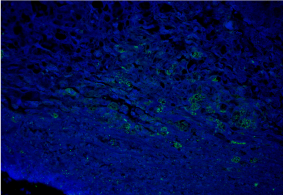

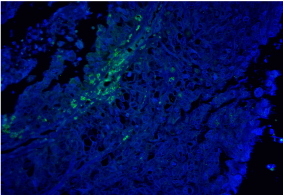


Control

60

p<0.05

n.s.

40

20

Antibiotics

0

Antibiotics+Ecoli

Antibiotics+Bt

Antibiotics+Acetic acid

CD86

CD163

60

p<0.05

n.s.

40

p<0.05

p<0.05

20

0
