# Supplementary material for: B. thetaiotaomicron-derived acetic acid modulate immune microenvironment and tumor growth in hepatocellular carcinoma
Source: Gut Microbes. 2024 Jan 25;16(1):2297846. doi: 10.1080/19490976.2023.2297846 (PMC10813637; doi:10.1080/19490976.2023.2297846)
Supplement: Figure S6.docx [file KGMI_A_2297846_SM9032.docx]

Figure S6

A B


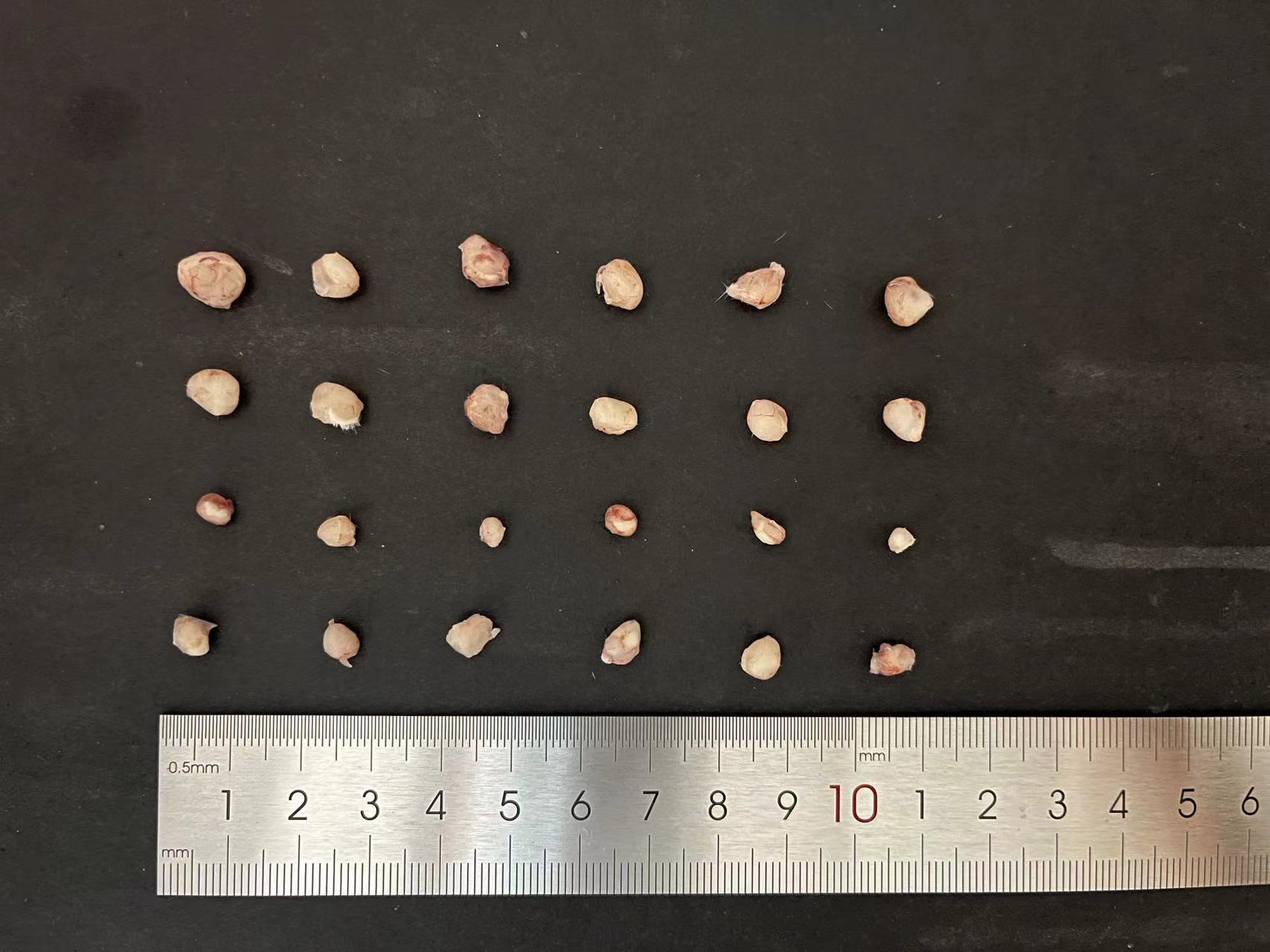
800

Contorl

Acetic acid+C646

B. thetaiotaomicron Acetic acid

Contorl Acetic acid+C646

B. thetaiotaomicron

Acetic acid

600

400

Tumor volume **（** mm 3**）**

200

0

6 9 12 15 18 21 24 27

Days
