# Supplementary material for: B. thetaiotaomicron-derived acetic acid modulate immune microenvironment and tumor growth in hepatocellular carcinoma
Source: Gut Microbes. 2024 Jan 25;16(1):2297846. doi: 10.1080/19490976.2023.2297846 (PMC10813637; doi:10.1080/19490976.2023.2297846)
Supplement: Figure S4.docx [file KGMI_A_2297846_SM9030.docx]

# Figure S4

A B

1.5

1.5

# C D

ACC1

1.0

p<0.05

0.5

ACSL1

0.0

1.0

0.5

FASN

0.0

TDM 2.5

2.0

Relative expression of mRNA

1.5

1.0

0.5

n.s.

p<0.05

ACC1


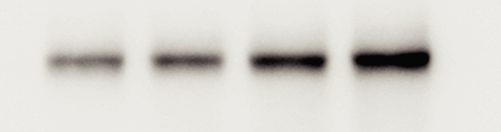

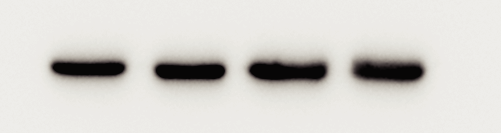


GAPDH

0.0

E

TDM

Relative expression of mRNA

Relative expression of mRNA

CD86

n.s.

p<0.05 p<0.05

NOS2

n.s.

p<0.05 p<0.05

F

CD163 ARG1

p<0.05 p<0.05

p<0.05 p<0.05

Relative expression of mRNA

2.5

Relative expression of mRNA

2.5

1.5

n.s.

1.5

n.s.

TDM

2.0

1.5

1.0

0.5

0.0

2.0

1.5

1.0

0.5

0.0

1.0

0.5

0.0

1.0

0.5

0.0

CD86

NOS2

CD163

ARG1 GAPDH


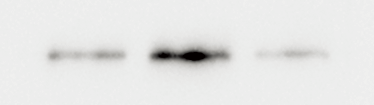

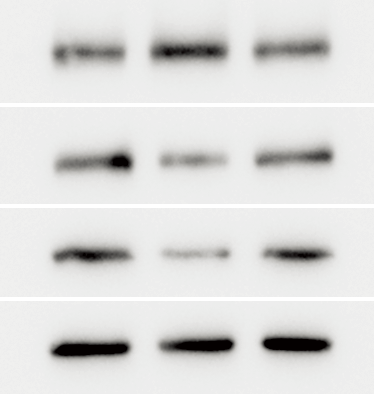


G


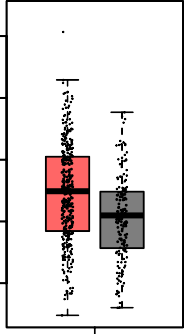


●

●

●

● ● ●

●

●

●

●

● ●

● ● ●

●

●

●

●●

●

●

● ● ●

● ● ●

●

●

●

● ●●

●

●● ●●

● ●● ●

●

● ● ●

● ● ●●

●● ●

●

●

●● ● ● ● ●●

● ●

● ● ●

●

● ● ●●

●

● ● ●

●

● ● ● ●

●●● ●●

●

● ● ●

●● ●●

●

●● ●● ● ● ●●

● ● ●

● ● ● ●●

● ● ●

● ● ● ●

● ●● ●

●● ●● ● ●

●● ●●

●

●

● ●

● ●

●

●

●

● ●

● ●

●

●

● ● ●● ●

● ● ●

● ●

● ●

● ●

● ● ● ●

● ●

● ●●

●

●

● ● ●

●● ●

● ●

●

● ● ●

● ● ●● ●

●● ●●

● ●●

●● ●● ●

●

●

●

● ● ●● ●

● ●

● ●● ●●● ●

●● ● ●

●

●

● ●● ●

● ● ●● ●

● ●

●● ● ●

● ● ● ●●

● ● ● ●●

● ●

● ●

● ●

● ●

● ●●

●

● ● ●

●

●● ● ●●

●● ● ●

● ●

●● ● ●

● ● ●

●

●

●● ● ● ●

● ●

● ●

●

● ● ● ●

● ●

●

●● ● ●

●● ●

●

● ●

●

● ●

● ● ● ●

●

●

● ●

●

●

● ●

●●● ● ●

●

● ● ● ●

● ●

●

●

● ● ●

●

● ●

●

●

● ●

● ● ●

●●

●

●

●

● ●

● ●

● ●

●

●

●

●

● ●

●● ●

●

●

●

●

●

● ●

●

●

●

●

●

● ●

●

ACC1 Expression-kig2(TPM+1)

4

5

6

LIHC (num(T)=369; num(N)=160)

2

3
