# Supplementary material for: B. thetaiotaomicron-derived acetic acid modulate immune microenvironment and tumor growth in hepatocellular carcinoma
Source: Gut Microbes. 2024 Jan 25;16(1):2297846. doi: 10.1080/19490976.2023.2297846 (PMC10813637; doi:10.1080/19490976.2023.2297846)
Supplement: Supplementary Table 1.docx [file KGMI_A_2297846_SM9029.docx]

| Gene Symbol | Forward | Reverse |
| --- | --- | --- |
| ACACA(ACC1) | ATGTCTGGCTTGCACCTAGTA | CCCCAAAGCGAGTAACAAATTCT |
| ACSL1 | CCATGAGCTGTTCCGGTATTT | CCGAAGCCCATAAGCGTGTT |
| FASN | AAGGACCTGTCTAGGTTTGATGC | TGGCTTCATAGGTGACTTCCA |
| CD8A | ATGGCCTTACCAGTGACCG | AGGTTCCAGGTCCGATCCAG |
| GZMB | CCCTGGGAAAACACTCACACA | GCACAACTCAATGGTACTGTCG |
| IFNG | TCGGTAACTGACTTGAATGTCCA | TCGCTTCCCTGTTTTAGCTGC |
| TNF | GAGGCCAAGCCCTGGTATG | CGGGCCGATTGATCTCAGC |
| CD86 | CTGCTCATCTATACACGGTTACC | GGAAACGTCGTACAGTTCTGTG |
| NOS2 | CCCCCAGCCTCAAGTCTTATT | CAGCAGCAAGTTCCATCTTTCAC |

Supplementary Table 1.The primers used for the tissue PCR
