# Supplementary material for: B. thetaiotaomicron-derived acetic acid modulate immune microenvironment and tumor growth in hepatocellular carcinoma
Source: Gut Microbes. 2024 Jan 25;16(1):2297846. doi: 10.1080/19490976.2023.2297846 (PMC10813637; doi:10.1080/19490976.2023.2297846)
Supplement: Figure S5.docx [file KGMI_A_2297846_SM9028.docx]

# Figure S5

A

## TDM 4

p<0.05

## BMDM 3

# B

## TDM 3

p<0.05

## BMDM 3

3

Pan-acylation

Pan-acylation

2 2 2

Anti-AC

Anti-AC

2

1 1 1 1

0 0 0 0

E coli Bt

p<0.05

E coli Bt

E coli Bt

p<0.05

p<0.05

p<0.05

E coli Bt

# C

## TDM 2.0

p<0.05 p<0.05 p<0.05

## BMDM 2.0

1.5 1.5

H3K27ace

H3K27ace

1.0

0.5

1.0

0.5

0.0 0.0

# D

## TDM

6

p<0.05

p<0.05 p<0.05

p<0.05

p<0.05

5

p<0.05

p<0.05 p<0.05

1.5

p<0.05

p<0.05

p<0.05

p<0.05

1.5

p<0.05

4

CD86

2

4

3

NOS2

2

1

1.0

0.5

CD163

1.0

0.5

ARG1

0

0

0.0

0.0

# E

p<0.05

p<0.05

p<0.05

## BMDM

p<0.05

p<0.05

p<0.05 p<0.05

5

4

CD86

3

2

1

0

4

3

p<0.05

p<0.05

p<0.05 p<0.05

NOS2

2

1

0

1.5

1.0

CD163

0.5

0.0

p<0.05

1.5

1.0

ARG1

0.5

0.0

p<0.05

p<0.05

p<0.05

p<0.05

p<0.05

p<0.05

p<0.05

p<0.05
